# Host-Parasite Relationship in Cystic Echinococcosis: An Evolving Story

**DOI:** 10.1155/2012/639362

**Published:** 2011-10-31

**Authors:** Alessandra Siracusano, Federica Delunardo, Antonella Teggi, Elena Ortona

**Affiliations:** ^1^Dipartimento di Malattie Infettive, Parassitarie e Immunomediate, Istituto Superiore di Sanità, Viale Regina Elena 299, 00161 Roma, Italy; ^2^Dipartimento di Biologia Cellulare e Neuroscienze, Istituto Superiore di Sanità, Viale Regina Elena 299, 00161 Roma, Italy; ^3^Dipartimento di Malattie Infettive e Tropicali, Ospedale Sant'Andrea, Sapienza-Università di Roma, Via di Grottarossa, 00189 Roma, Italy

## Abstract

The larval stage of *Echinococcus granulosus* causes cystic echinococcosis, a neglected infectious disease that constitutes a major public health problem in developing countries. Despite being under constant barrage by the immune system, *E. granulosus* modulates antiparasite immune responses and persists in the human hosts with detectable humoral and cellular responses against the parasite. *In vitro* and *in vivo* immunological approaches, together with molecular biology and immunoproteomic technologies, provided us exciting insights into the mechanisms involved in the initiation of *E. granulosus* infection and the consequent induction and regulation of the immune response. Although the last decade has clarified many aspects of host-parasite relationship in human cystic echinococcosis, establishing the full mechanisms that cause the disease requires more studies. Here, we review some of the recent developments and discuss new avenues in this evolving story of *E. granulosus* infection in man.

## 1. Introduction

Human immune system has evolved specialized mechanisms and cell populations to protect us from the full spectrum of pathogens that poses very different problems for the immune system. Helminthes have developed complex evasion strategies and, when the immune response falls short, it may be necessary for the host to enter a damage limitation state, accommodating infection in order to minimize pathology. Parasite immune evasion mechanisms themselves depend on a form of molecular dialogue between pathogen and host and, in turn, many parasites depend on host molecular signals for their development [1].

During cystic echinococcosis (CE) the host-parasite relationship is interactive and the outcome of infection depends on the balance achieved by the combination of the different variables involved with the host immunity and the *E. granulosus* avoidance strategies [2]. An understanding of the biological events occurring during infection is necessary to visualize the diverse immune stimuli to which the parasite subjects the host and to define diagnostic and therapeutic tools. We discuss in detail these topics in this review. 

## 2. *E. granulosus* Epidemiology

CE, a chronic endemic helminthic disease caused by infection with metacestodes (larval stage) of the tapeworm *E. granulosus*, is one of the most widespread zoonotic diseases in humans in both developing and developed countries [3]. Recently, the World Health Organization included echinococcosis as part of a Neglected Zoonosis subgroup for its 2008–2015 strategic plans for the control of neglected tropical diseases [4, 5]. The distribution of *E*. *granulosus* is worldwide, with only a few areas such as Iceland, Ireland, and Greenland believed to be free of autochthonous human CE [6]. CE is prevalent in countries of the temperate zones, including South America, the entire Mediterranean region, Russia, central Asia, China, Australia, and parts of Africa [3, 7–9]. In the USA, most infections are diagnosed in immigrants from countries in which echinococcosis disease is endemic. Sporadic autochthonous transmission is currently recognized in Alaska, California, Utah, Arizona, and New Mexico [10].

*E. granulosus* comprises a number of forms that exhibit considerable genetic variation [11]. Ten strains of *E. granulosus* (G1–10) have been described with molecular biology techniques using mitochondrial DNA sequences [12]. These include the common sheep strain (G1), a Tasmanian sheep strain (G2), two bovine strains (G3 and G5), a horse strain (G4), a camel strain (G6), a pig strain (G7), a cervid strain (G8), a Poland swine strain (G9) [12], and an Eurasian reindeer strain (G10). Recent molecular re-evaluation of *Echinococcus* species strongly suggests that *E. granulosus* is an oversimplified species. The genotypes G1 to G5 have been reclassified into *E. granulosus sensu stricto* (G1 to G3), *E. equinus* (G4), and *E. ortleppi* (G5). The genotypes G6 to G10 and the lion strain of *E. granulosus* (formerly *E. felidis*) have to be re-evaluated [13]. The sheep strain (G1) has a worldwide geographical distribution, specifically it is widely spread in North Africa and has a natural circulation in some European countries such as Italy [14]; this strain is commonly associated with human infections. G2 strain is geographical distributed in Tasmania and Argentina but there are recent observations of the emergence of the presence of this strain also in some endemic European countries as Bulgaria, Italy, France, Portugal, and Spain. G3 strain has a major distribution in Asia and in more endemic European countries. The “cervid” genotype (G8) cycle involves wolves, dogs, mooses and reindeers [12]. 

Despite the increasing epidemiological reports, available information on CE is still incomplete and is insufficient to assess properly its world epidemiology. CE importance tends to be underestimated due to underreporting and to the lack of compulsory notification. To note, the reporting of incidental cases is mandatory in most of the EU member countries except Denmark and Italy [15]. These facts strongly recommend the convenience of maintaining and/or intensifying the control measures currently in place in order to consolidate the progress achieved and to avoid the recrudescence of the disease. 

## 3. *Echinococcus* Metacestodes

### 3.1. *E. granulosus* Biology

The complex cycle of the parasite can explain the intricate host-parasite relationship. *E. granulosus* is a small tapeworm (rarely exceeding 7 mm in length) that lives firmly attached to the mucosa of the small intestine in definitive hosts, usually dogs, where the adult-stage reaches sexual maturity within 4 to 5 weeks. This is followed by the shedding of gravid proglottids (each containing several hundred eggs) and/or of released eggs in the feces of definitive hosts. After being ingested by the intermediate host, eggs release embryos (oncospheres) that penetrate the gut wall, travel via blood or lymph, and are trapped in the liver, lungs, and other sites where cystic development begins. This process involves transformation of the oncospheral stage to reach the metacestode stage. 

*E. granulosus* typically develops as a large unilocular, turgid cyst, which grows through an increase in diameter from less than 1 to 5 cm each year. This general structure can be thought to allow a permanent low ratio between total parasite cellular volume and host-exposed area, through linear growth that can exceed three orders of magnitude. Hydatid cyst is usually surrounded by a host-derived collagen capsule (adventitial layer), but can also been circled by host inflammatory cells. Metacestode (hydatid cyst) is bounded by the hydatid cyst wall, which comprises an inner cellular layer (germinal layer) and an outer protective acellular layer (laminated layer). The germinal layer (GL) gives rise towards the cyst cavity to cellular buds that upon vesiculation become brood capsules, and in turn bud towards their inside to generate protoscoleces. The GL exposes towards the outside the apical plasma membrane of its syncytial tegument, which carries truncated microtriches. The GL has additional, nonsyncytial cell types, including muscle, glycogen-storage, and undifferentiated cells. Towards the cyst cavity, there is neither a syncytial organization nor junctional complexes between cells, so that the intercellular fluid of the germinal layer is apparently continuous with the cyst/vesicle fluid [16, 17].

In spite of being widely considered the crucial element of host-parasite interfaces, the laminated layer (LL), a structure only found in the genus *Echinococcus*, is poorly understood. In fact, it is still often called “chitinous,” “hyaline,” or “cuticular” layer, or said to be composed of polysaccharides. However, over the past few years the LL was found to be comprised of mucins bearing defined galactose-rich carbohydrates, and accompanied by calcium inositol hexakisphosphate deposits. A recent review discusses the architecture and biosynthesis of this unusual structure [18]. The cyst cavity is filled with hydatid cyst fluid (HCF) that is the main factor responsible for the antigenic stimulation. The hydatid liquid is clean and clear, “as well as the clean water from its natural source,” containing secretions from both the parasite and host and all the elements from the “inner wall” of the cyst, named hydatid sand [19]. It has an identical composition to that of the host's serum (Na, K, Cl, CO_2_, a density between 1.008 and 1.015, alkaline pH) and some specific proteins that confer antigenic properties such as Ag5 and AgB. 

### 3.2. *E. granulosus* Natural History

The natural history of *E. granulosus *cysts and its clinical implications comprises various developmental stages. The initial stage, primary infection, is always asymptomatic. During this stage, small (<5 cm) well-encapsulated cysts develop in organ sites, where they persist inducing no pathologic consequences. In humans, the hydatid cysts are localized in approximately two-thirds of cases in the liver and in about 20% in the lungs, and less frequently in the kidneys, spleen, heart, and bone. Some 20–40% of patients have multiple cysts or multiple organ involvement. After an undefined incubation period lasting months or years, if cysts exert pressure on adjacent tissue and induce other pathologic events, the infection may became symptomatic. Because hydatid cysts grow slowly, the host often tolerates it remarkably well. Patients with CE may come to clinical attention only when a large cyst mechanically alters body function, when allergic phenomena or other miscellaneous symptoms such as eosinophilia develop, or when the cyst accidentally ruptures thus triggering acute hypersensitivity reactions. Cysts or a cystic mass may also be discovered by chance during body scanning or surgery, or for other clinical complications [19]. During the outcome of the infection, several events can occur into the cyst: the death of the parasite due to dysfunction of the GL (detachment or aging), the “cyst's wall” fissure due to detachment of membranes or micro traumatisms, the transformation of scoleces into vesicles (vesiculation). These new vesicles, called offspring or “daughter” vesicles, live into the hydatid fluid and have the same constitution as well the same mission of the mother vesicle and occasionally form within larger cysts. Therefore, in this way, protoscoleces may develop into either a new cyst or an adult parasite. 

The extensive variation at the genetic level may influence *E. granulosus* life development rate, cycle patterns, host specificity, antigenicity, transmission dynamics, sensitivity to chemotherapeutic agents, and pathology with important implications for the design and development of vaccines, diagnostic reagents and drugs. To note, human infection with G8 strain presents a predominantly pulmonary localization, slower and more benign growth, and less frequent occurrence of clinical complications than reported for other strain genotypes [10]. Zhang and McManus have recently extensively reviewed a detailed account of genetic variation in *Echinococcus* and its implications [20].

## 4. *E. granulosus* Antigens

Since the 1960s, research on CE has been focused on the identification of immunologically important proteins, especially potential immunodiagnostic or vaccine candidates. Because of the expression of different antigens during the different developmental stages, the human host responds independently to antigenic stimuli of the invading oncosphere, the metacestode in transformation from the oncosphere, and finally, the mature metacestode (larvae) [2]. *E. granulosus* immunology has been divided into an “establishment” phase during which the parasite is most susceptible to host effectors, and an “established metacestode” phase during which the parasite elicits chronic disease. In the early stages of echinococcal development, cellular responses may play a crucial role in protection against infection [21]. 

Older studies reported that the oncospheres stimulate a strong immunity to a challenge infection [22]. Strong antibody responses against purified oncosphere proteins have been reported also in sera from experimentally infected sheep [23]. Most recent experiments in mice showed that a second oncospheral challenge 21 days after the primary infection with *E. granulosus* produced very high levels of protection but with a very low antibody response [24]. Therefore, because the oncosphere is known to be associated with the protective immune response, understanding the mechanisms whereby protective antibodies against the oncosphere act, is of fundamental importance in developing highly effective vaccine against *E. granulosus* [25]. The results of Heath and Lawrence [23] settled the basis for the development of the Eg95 vaccine in ruminants [26, 27]. 

The LL, an insoluble and unusual biological structure, is the crucial element of host-parasite interface in larval echinococcosis. Because of its massive carbohydrate-rich structure and resistance to proteolysis, it contains few T-cell epitopes and abundant T-independent anti-carbohydrate antibodies. Consequently, the innate immunity induces a noninflammatory response and the adaptative immunity induces a humoral response characterized by low-avidity antibodies specific for *α*-galactose [28]. The history of LL represents an example of our evolving knowledge in the immunological mechanisms that *E. granulosus* takes to survive in the host. It has been fascinating to arrive at explanations for observations that lay forgotten in papers published decades back [29, 30]. In particular, in 1974, we have observed that sera from patients with pulmonary cyst localization presented antibodies against a glycoprotein antigen (*α*-galactosyl residue) isolated from the hydatid membrane. This antigen showed a high P_1_ blood group activity thus suggesting an intriguing role for the hydatid membrane in the host-parasite relationship [30]. Later, P1 blood antigen has been also identified in protoscolex [31]. A recent review describes in depth the modern studies on the biochemistry of LL that allowed a more informed analysis of its immunology [28]. The major immunodiagnostic protein antigens are present in HCF [32]. However, if T-independent anti-carbohydrate responses are included, the laminated layer may instead be the major source of antigens [28]. The GL of the cyst is a barrier against immune competent cells of the host. It is generally thought that damages in the GL, like fissures or rupture, induce an antigenic stimulation. When this antigenic stimulation occurs, there is a continuous elevation of the immunologic values for an indeterminate time. This elevation also happens after the cyst manipulation (surgery, puncture, etc.) [33]. 

Extensive studies have focused on hydatid fluid antigens that still represent the main antigenic source for hydatid disease diagnosis. At the present time, despite the large number of studies, the parasitic antigens present in HCF that have major immunodiagnostic value in detecting *E. granulosus *are antigen 5 (Ag5) and antigen B (AgB) [34, 35]. Native Ag5, a 400 kDa thermolabile glycoprotein produces two subunits at 55 and 65 kDa in sodium-dodecyl sulphate-polyacrylamide gel electrophoresis (SDS-PAGE) under nonreducing conditions and two subunits at 38/39 and 22–24 kDa under reducing conditions [36–38]. The biological role of Ag5 is almost completely unknown, although its elevated concentration in HCF suggests a relevant function in the development of the metacestode. The 38/39 kDa component with phosphorylcholine epitopes may be responsible for a large proportion of cross-reactions with sera from patients infected with nematodes, cestodes, and trematodes [36–39]. The 38 kDa subunit is closely related to serine proteases of the trypsin family, but has no detectable proteolytic activity [40]. Studies by sequencing of the N-terminal fraction of the 38 kDa subunit revealed a single amino acid sequence with alternative residues at some positions, demonstrating that Ag5 is present in different isoforms [41]. Regarding the 22 kDa subunit, the heparan sulphate proteoglycans and calcium-binding sites found in this component seem to provide binding targets for the Ag5 molecule [40]. These would target the antigen and ensure its localization in the host tissue surrounding the metacestode, or otherwise, the mucosal epithelium of the *E. granulosus* definitive host. Ag5 has been widely used in the serodiagnosis of human CE, particularly by means of the identification of a precipitation line (arc 5) in immunoelectrophoresis assays [32]. González-Sapienza et al., identified and cloned a metacestode-specific component (named P29) immunologically related to, but distinct from, Ag5 [42]. This finding would imply that much of the information derived from studies carried out using antibodies to Ag5 could be equivocal because of the cross-reactivity between both Ag5 and P29 [43]. 

Native AgB, a 160 kDa thermostable lipoprotein, produces three main subunits at 8/12, 16, and 20 kDa in SDS-PAGE under reducing and nonreducing conditions as well as other mass subunits, probably polymers of the 8/12 kDa subunit [44]. The 8/12 kDa subunit induces a good humoral and cellular response [45]. Even though the 8/12 kDa subunit of AgB is cross-reactive in a high percentage of patients with alveolar echinococcosis sera and in a small percentage of patients with cysticercosis, native AgB is of high immunodiagnostic value [32, 39, 46]. The oligomeric organisation of the *E. granulosus* AgB (EgAgB) was further investigated by González et al. [47], who analysed the subunit composition of EgAgB in HCF by comparing the amino acid sequence of tryptic peptides isolated from the 8, 16, and 24 kDa subunit bands of native EgAgB with that of the 8 kDa subunit monomers and found that the 8 kDa band contained at least two components, which constituted the building blocks of the higher molecular weight subunit bands. Further progress towards characterising AgB came from experiments using DNA cloning [44, 48, 49]. Using this technique, Shepherd et al. [50] reported a cDNA clone encoding the carboxy-terminal of the 12 (8) kDa subunit of antigen B and Frosch et al. [51] described its complete sequence (AgB/8 or EgAgB8/1). Nucleotide variations are present at a conserved position between AgB/8 cDNA sequences from different isolates, indicating that this gene is polymorphic. Others later isolated a cDNA clone coding for a second 8 kDa subunit of AgB (EgAgB8/2) [52]. Specific antibodies against both antigens recognized all AgB bands in western blot, and peptide sequencing revealed that both antigens are components of the native AgB subunits [47]. Together these results show that AgB is made up of subunits encoded by at least two different genes. Molecular studies now show that *E. granulosus* AgB is encoded by a multigene family having at least five gene loci (B1–B5), each one consisting of several minor variants that phylogenetic tools grouped into two clusters: EgAgB1/B3/B5 and EgAgB2/B4 [51, 53–56]. A more recent phylogenetic analysis failed to discriminate between the isoforms EgAgB3 and EgAgB5 [57]. The putative protein isoforms encoded by the five EgAgB genes differ in amino acid sequence (44–81%). Switching from one isoform to another could be among the mechanisms parasites use to evade the host's immune response and to modulate periparasitic inflammatory reactions [55]. Recently, Muzulin et al. showed that *E. granulosus* strains differ in the type of genomic and transcribed EgAgB sequences, reinforcing previous evidence that the AgB gene family is highly polymorphic [49]. How this variation affects the way each strain adapts to its specific intermediate host, and whether it influences AgB's potential as a diagnostic tool remain matters for future studies. In contrast with previous data, showing that *E. granulosus* strains differ in the types of genomic and transcribed EgAgB sequences, Zhang et al. found that the EgAgB gene family comprises at least ten unique genes, each of them was identical in both larval and adult *E. granulosus* isolates collected from two different continents [58]. DNA alignment comparisons with EgAgB sequences deposited in GenBank databases showed that each gene in the gene family is highly conserved within *E. granulosus*, which contradicts previous studies claiming significant variation and polymorphism in EgAgB. Quantitative PCR analysis revealed that the genes were differentially expressed in different life-cycle stages of *E. granulosus* with EgAgB3 expressed predominantly in all stages. Finally, Chemale et al. [59] characterising the properties of native EgAgB to bind hydrophobic ligands and comparing the activity of two of the 8 kDa subunit monomers (rEgAgB8/1 and rEgAgB8/2), found that the hydrophobic ligand binding properties of EgAgB differ from the helix-rich hydrophobic ligand binding properties displayed by proteins from other cestodes. Because many of these proteins are immunogenic and some are involved in lipid detoxification, transport, and metabolism with their fatty acid binding properties, AgB could be involved in the process of parasite survival in host microenvironment.

Similar to *E. granulosus*, AgB also exists in the cyst fluid of *E. multilocularis* and AgB genes are expressed in a developmentally regulated manner in *E. multilocularis* vesicles, protoscoleces, and immature adult worms [44]. 

In the 1990s it had become apparent that the new techniques in molecular biology offered a new approach to overcome some problems and several recombinant antigens have been produced and used as molecular tools in the immunodiagnosis of CE [60] (Table 1). In a series of molecular studies, we screened an *E. granulosus* cDNA library with IgE from patients with CE who had acute cutaneous allergic manifestations and we identified three conserved constitutive proteins: EgEF-1 *β*/*δ*, EA21, and Eg2HSP70 [61–64].

Later, we screened an *E. granulosus* cDNA library with IgG4 from patients with active disease and with IgG1 from patients with inactive disease. By screening with IgG4 from patients with active disease, we obtained two proteins. The first is present on the protoscolex tegument and on the GL of cyst wall (EgTeg) and the second protein has 19.0 kDa (Eg19) [65, 66]. By screening the *E. granulosus* cDNA library with IgG1 from patients with inactive disease, we obtained EgTPx [67].

## 5. *E. granulosus* and Antibody Responses

There are extensive data on immune responses against the hydatid cyst both from studies on patients with *E. granulosus *infection and from experimentally infected animals [22]. The established parasite produces significant quantities of molecules that modulate the immune responses and these include both humoral and cellular immune response against the parasite. 

Although the data are limited, there is, nevertheless, clear evidence from experiments with animals challenged with *E. granulosus* eggs or oncospheres that infected hosts produce significant immune responses, including antibodies and T cell responses generated by lymphocytes. 

The earliest IgG response to oncospheral antigens appears after 11 weeks in mice and sheep challenged with eggs or oncospheres of *E. granulosus* [23]. These anti-oncospheral antibodies play a major role in parasite killing and are central to the protective immune response against *E. granulosus*. 

Numerous studies demonstrated that *E. granulosus *HCF induces a strong humoral response in humans. Even if sera from patients with CE contain abundant circulating IgG, IgM, and IgE antibodies to *E. granulosus* antigens, none of these antibodies is associated with protection [75]. Because IgG antibodies, that retain floating levels for many years even after “cure,” cannot be considered as immunological markers of the outcome of therapy, the analysis of IgG subclass, that vary during the outcome of the disease, has been considered for a long time useful in follow-up [18]. In contrast with these results, we demonstrated that the expression of the various IgG isotypes remained practically unchanged over a long-term follow-up, but antibody levels before therapy differed in the patients grouped according to the outcome of chemotherapy. IgG isotype expression differed also in its HCF and AgB binding profiles. Hence, although IgG isotypes cannot be considered as immunological markers of the outcome of chemotherapy, we concluded that they might be a useful guide to the clinical management of CE [76]. Recently Pan et al. demonstrated that because the expression of AgB2 declines with progression of the disease, this antigen is a suitable immunological marker for detection, diagnosis, and progression of the disease [77]. Given that the first studies of IgG subclass antibody responses in advanced human CE indicated a switch from predominant IgG1 response to IgG4 in CE patients with progress disease, the peculiar role of IgG4 during CE has been extensively studied and IgG4 actually are considered as immunological markers during CE [78]. IgG4 is a subclass associated with prolonged, chronic infection, that is neither cytophilic nor complement fixing, is nonfunctional, and binds weakly to receptors for the Fc portion of immunoglobulins, it may help the parasite to evade the host immune response [79]. Moreover, parasite-specific IgG4 antibodies can inhibit IgE-mediated degranulation of effector cells reducing allergic pathology in the host [80]. In agreement with these studies, we found that albendazole-treated patients, who exhibited a good therapeutic and clinical response to treatment, had significantly lower levels of serum IgG4 antibodies, than poor responders or nonresponders whereas IgG1 antibody levels showed a reverse trend [81, 82]. Later we confirmed the presence of higher IgG4 and IgE in patients with progressive disease and higher IgG1 and IgG3 in patients with stable disease [76].

## 6. *E. granulosus* and Cytokine Induction

A key question is how *E. granulosus* that encounter the immune system can influence the differentiation decision. Th1 and Th2 cells are not precommitted phenotypes but rather, represent endpoints of a multistep differentiative process, whereby a common precursor population acquires a distinct cytokine secretion profile [83]. During CE, the evidence concerning antibody levels of IgG4 and IgE isotypes and frequent eosinophilia, suggested that the immune response to established *E. granulosus* infection is Th2 dominated and that *Echinococcus* antigens modulate polarized T-cells. Immunological studies conducted in our laboratory, showing high *in vitro* production of parasite antigen-driven IL-4, IL-5, IL-6, IL-10, and IFN-*γ* by peripheral blood mononuclear cells (PBMC) isolated from patients with CE, confirmed that the human immune response to *E. granulosus *infection is predominantly regulated by Th2 cell activation but also by the Th1 (or Th0) cell subset. [81, 82]. Data obtained in *E. granulosus* experimental infection supported the hypothesis that early IL-10, secreted by B cells in response to nonproteic antigens, may favor parasite-survival and the establishment of a polarized type-2 cytokine response [84]. Recent findings suggested that IL-4/IL-10 impairs the Th1 protective response and allows the parasite to survive in hydatid patients [85]. Experimental studies in mice supported the possible local immunosuppression mediated by IL-10 and TGF-*β* as possible mechanism that helps the parasite in escaping the host cell-mediated response [86].

To note, the probable immune-suppressing effects of TGF-*β* (and regulatory T cells) have been shown to be present in *E. multilocularis* experimental infection. Intraperitoneal murine *E. multilocularis* infection induces differentiation of TGF-*β*-expressing dendritic cells (DCs) that remain immature and modulates peritoneal CD4^+^ and CD8^+^ regulatory T-cell development [87]. 

Evidences highlighting crucial role of cytokines in the host-parasite relationship come from studies on parasite-driven cytokine production in a large number of albendazole-treated patients with CE. PBMC from patients who responded to chemotherapy produced high amounts of IFN-*γ* (Th1 derived) whereas PBMC from patients who did not respond produced IL-4 and IL-10 (Th2 derived). We later confirmed this finding in a molecular study by detecting IL-12 p40 mRNA in 86% of successfully treated patients at the end of chemotherapy. PBMC from patients in whom therapy failed, expressed weakly IL-4 mRNA before therapy, and strongly thereafter; PBMC from patients who responded to therapy expressed higher IFN-*γ* and TNF-*α* mRNA values than patients who did not [88]. Finally, T cell lines from a patient with an inactive cyst had a Th1 profile whereas T cell lines derived from patients with active and transitional cyst had mixed Th1/Th2 and Th0 clones [89]. Since PBMC from seronegative patients produced no parasite antigen driven-IL-5 and scarce IL-4 and IL-10, we suggested that during CE the seronegativity occurs because host or parasite factors or both preclude Th2 cell activation thus limiting or preventing production of IL-5, the cytokine that has a critical role in immunoglobulin expression [90].

Collectively our data indicated that in CE a strong Th2 response correlates with susceptibility to disease (active cyst) whereas a Th1 response correlates with protective immunity (inactive cyst) and that Th1 and Th2 responses coexist. 

The role of DCs in the immunity of CE and in the host-parasite relationship has been recently evaluated. Inflammatory mediators or microbial agents promote the migration of DCs into the secondary lymphoid organs. As they migrate, DCs mature, lose their Ag-capture ability, and gain an increased capacity to prime T cells. DC-parasite interactions are pivotal in triggering and regulating parasite-induced immunity. DC function is itself modulated during parasitic infection for the mutual benefit of the host and of the parasite [91, 92]. *E. granulosus* hydatid fluid modulates DC differentiation and cytokine secretion [93]. We have demonstrated that *E. granulosus* hydatid fluid impairs monocyte precursor differentiation into immature DCs rendering them unable to mature when stimulated with lipopolysaccharides. The parasite modulates also sentinel DC maturation, priming them to polarize lymphocytes into Th2 cells [94]. Collectively, these cellular findings establish that *E. granulosus* can directly influence the components of host cellular response, T lymphocytes, and DCs. 

## 7. *E. granulosus* and Immune-Modulating Molecules

Because *E. granulosus* inhabits immunocompetent hosts for prolonged periods it is not surprising that it should possess modulator molecules that remodel host responses to enhance its survival. AgB is the principal *E. granulosus* immune-modulant antigen [45]. Because it can modulate both innate and adaptive host immune responses, AgB plays a prominent role in the immunomodulatory mechanisms that *E. granulosus* uses to develop, progress, and cause chronic disease [2]. To survive in host tissues the parasite must be able to adapt metabolically to the host microenvironment, and plentiful AgB in HCF probably guarantees parasite survival. A large amount of data suggests that AgB directly immunomodulates the host immune response by inhibiting PBMC chemotaxis and indirectly by skewing the Th1 : Th2 cytokine ratio towards a preferentially Th2 polarization associated with chronic CE disease. 

The 12 kDa subunit of AgB is a serine protease inhibitor with strong chemoattractant activity and with the ability to inhibit human neutrophil chemotaxis without altering either random migration or oxidative metabolism [50, 95]. In agreement with the negative immunomodulatory role suggested for AgB on human neutrophils, when accidentally released hydatid fluid activates neutrophils, AgB could act as an interference antigen allowing the released protoscoleces to develop into secondary cysts [96]. We investigated the role of AgB in acquired immunity by evaluating AgB-driven Th1 and Th2 cytokine production by PBMC from patients with CE [95–97]. Patients' PBMC stimulated with AgB produced IL-4, IL-13, and low IFN-*γ* concentrations, but did not produce IL-12. This Th2 polarization was more evident in patients with active disease, in whom the stimulus with AgB increased the imbalance observed in cultures from patients with inactive disease [89]. Finally, AgB modulates sentinel DCs maturation, priming those to polarize lymphocytes into an exclusive Th2 response that benefits the parasite (IL-4 expression). Our data offer a rationale for this polarization by showing that if AgB encounters immature DCs, it suppress IL-12p70 production by inducing the immunoregulatory cytokine IL-10. AgB reduces lipopolysaccharide-induced production of IL-12p70 but not of IL-6, providing further evidence that it actively modulates DC responsiveness in a manner favouring a Th2 outcome [94].

In a series of molecular studies, we screened an *E. granulosus* cDNA library and we identified constitutive proteins (EgEF-1 *β*/*δ*, EA21, Eg2HSP70, EgTeg, Eg19, and EgTPx) that appear to have immunomodulant propriety. The EgEF-1 *β*/*δ* intervenes in immunomodulation because it continues to be released into the hydatid fluid after the protoscoleces degenerate; in fact, we found a higher percentage of antibodies specific against EgEF-1*β*/*δ* in patients with CE who had inactive cysts than in patients with active cysts [61, 62]. Also we found that a high percentage of sera from patients with CE without allergic manifestations had IgG4 antibodies specific to EA21 whereas patients with allergic manifestations showed IgE specific to EA21 we suggested that in CE, as in other parasitic diseases, IgG4 apparently acts to block pathogenic processes, minimizing severe pathology in the host [63]. Regarding Eg2HSP70, this antigen seems to elicit IL-4 production not through its intrinsic ability but by strengthening, the generalized Th2 polarization previously established [64]. 

EgTeg is an immunomodulatory molecule that, as AgB, contributes to chronic infection by inhibiting chemotaxis and inducing IL-4 and IgG4 [65]. Regarding Eg19 reactivity, the percentage of total IgG-, IgG1-, and IgG4-positive sera were significantly higher in sera from patients with active disease and cyst in multiple sites than from patients with inactive disease and cyst in the liver. Because anti-Eg19 antibody concentration decreased over the course of treatment in sera from patients with cured disease, our data, confirming the presence of antigens inducing both IgG1 and IgG4 during active CE, suggest that Eg19 might be a marker of disease status [66]. 

EgTPx seems to have an unclear role in immunomodulation, further researches are necessary to clarify precisely how EgTPx intervenes in immune evasion and whether anti-EgTPx antibodies can be used to counteract larval survival and development [67]. In a recent review about the *E. multilocularis* parasite-host interplay, Gottstein and Hemphill described the protein and glycoprotein composition of the laminated layer and the E/S fraction, including Em2- and Em492-antigens, two metacestode antigen fractions that exhibit immunosuppressive or -modulatory properties [98]. An important molecule is the 14-3-3 protein family, small proteins (30 kDa), described and characterized in several parasites and mostly studied in *E. granulosus* and *E. multilocularis. *In a recent review, Siles-Lucas et al. have deeply described new data about this protein and its important implications in the parasite biology and immunology in the frame of the host-parasite relationship [99]. 

## 8. New Perspectives from Proteomic

The advent of proteomics techniques, applied to the analysis of the protein content of biological fluids, has significantly improved the identification and characterization of proteins from *E. granulosus *metacestode to use as potential new diagnostic and prognostic indicators. The use of related analytical techniques also offers the opportunity to gain information on regulation, via post-translational modification, and on elucidation of the protein expression profile in different parasite stages and in different disease stages. Moreover, proteomics will certainly play an important role in the study of changes in the protein expression levels of protoscoleces in response to external factors, such as anti-helmintic treatment, stress and in the comparative analysis of cysts from different hosts or between active and resting cysts [100, 101].

The lack of a complete sequenced genome and the presence of highly abundant host serum proteins prevented for long time the *E. granulosus* metacestode proteomic analysis. However, these negative factors have been at least in part compensated by the availability of a comprehensive *E. granulosus* EST database and by the use of an immunopurification approach to enrich samples with proteins from parasite origin, respectively. Therefore, the strategy of searching not only in the *E. multilocularis* EST database but also in the EST data available from other platyhelminthes allowed to extend the previously restricted overall repertoire of known proteins expressed and released by the *E. granulosus* metacestode [102].

Chemale et al. [100] reported for the first time the proteomic technique for identification of new proteins in *E. granulosus*. These authors, using a parasite-enriched fraction from a whole protoscoleces protein extract, identified and analyzed by MALDI-TOF-MS 100 prominent protein spots. They identified important proteins, such as actin, tropomyosin, paramyosin, thioredoxin reductase, antigen P-29, cyclophilin, and the heat shock proteins hsp70 and hsp20. Three different protein spots were identified as actins, and this data confirms previous results suggesting the existence of several actin genes in the *E. granulosus *genome [103]. 

More recently, Monteiro et al. [102] analyzed antigens of *E. granulosus* during infection of its intermediate bovine host. They used an immunoproteomic strategy joining immunoblot immune screening with proteome technologies involving 2-DE-PAGE and mass spectrometry for the identification of proteins. Parasite proteins were identified in different metacestode components (94 from protoscolex, 25 from GL, and 20 from HCF). The subsequent search for antigenic proteins by immunoblot resulted in the identification of many proteins recognized by cystic hydatid disease patient sera. As well as proteins previously identified as antigens (P-29, EgTPx, EgcMDH, HSP70, grp78, actin, calreticulin, tropomyosin, HSP20, and 14–3–3), the authors identified for the first time five antigens: enolase, GST, putative MVP protein, fructose-bisphosphate aldolase, and citrate synthase. Moreover, they found proteins that may contribute to immunoregulatory events such as paramyosin and tetraspanin, proteins contributing to the establishment of an *E. granulosus* chronic infection as AgB and EgTeg.

Aziz et al. [101] used a different proteomic approach: firstly, 1D SDS-PAGE gels were used to fractionate HCF and these were divided into thirty bands and subjected to LC-MS/MS after in-gel digestion. Secondly, a large quantity of HCF was analysed using peptide OGE and an LC-MS/MS protocol that incorporated an extended (2 h) LC step. Using these techniques, they were able to identify 130 protein constituents of HCF from three intermediate hosts of *E. granulosus*. Over forty parasite proteins were identified in HCF, the most abundant being AgB and Ag5, two known antigens. As in previous studies [91, 95], thioredoxin peroxidase and two isoforms of the low-density lipoprotein receptor were identified and these are likely to aid parasite survival by protecting against oxidative damage and in the uptake of sterols and fatty acids from the host, respectively. Other identifications included cyclophilin, ferritin, heat shock proteins, annexin A13, and cathepsin B.

We have exploited the classic immunoproteomic strategy to identify *E. granulosus* antigens distinctive of different stage of the disease [74]. Two-dimensional gel electrophoresis (2-DE) of HCF, followed by immunoblot analysis with sera from patients with distinct phases of disease enabled us to identify, by mass spectrometry, HSP20 as a potential marker of active CE. Immunoblot analysis revealed anti-HSP20 antibodies in a statistically significant higher percentage of sera from patients with active disease than in sera from patients with inactive disease. Anti-HSP20 antibody levels significantly decreased over the course of pharmacological treatment in sera from patients with cured disease, relative to sera from patients with progressive disease. This proteomic approach emphasizes the presence of a large number of antigenic proteins associate to parasite immune evasion during the development of the disease and highlights the difficulty in understanding the host-parasite relationship. 

## 9. Conclusions

**Figure 1:**
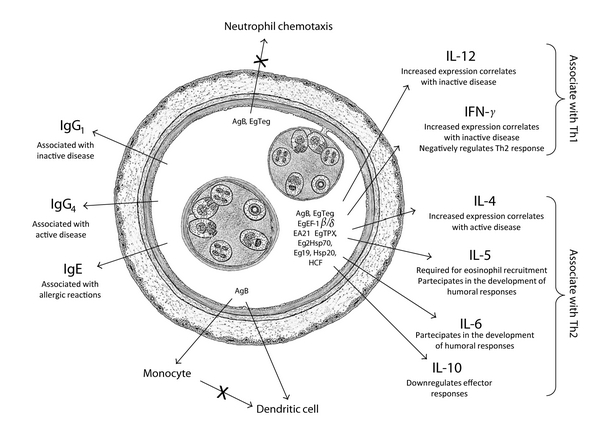
Major components of the immune response to hydatid cyst fluid in the host: *Echinococcus granulosus*-derived immune modulators and the main cytokines that regulate this response. Parasite-derived molecules as AgB, EgTeg, and EgEF-1*β*/*δ* could elicit a predominant Th2 activation whereas EgTPx and other HCF components can elicit a concomitant Th1/Th2 cell activation.

The hydatid cyst secretes and exposes numerous immunomodulatory molecules to the host's immune system. Throughout the past 30 years, experimental studies probing the immunobiology of *E. granulosus* have begun to uncover an evolving story in which parasite immunomodulating proteins actively interact with innate and adaptative human immune processes to reduce the impact of a host response (Figure 1). The natural history of cyst development indicates that each cyst is a story in itself and that significant efforts must be made to establish markers of cyst viability and of nature and intensity of immune response. 

Clinical proteomic looks like one of the most conceptually and scientifically sound ways of generating and exploiting new biological insights and technologies for the benefit of patients. Alternative strategies, such as generation of multiplex quantitative immunoassays, may need to improve diagnosis, classification, prediction of treatment response, and prognosis of CE disease. In conclusion, the *E. granulosus* story is not over yet, but continues.

## Figures and Tables

**Table 1:** Main* Echinococcus granulosus* antigenic molecules identified and characterized, and/or recombinantly expressed.

Antigen	Name	References
Antigen 5	Ag5	Capron et al. [36]

Antigen B	AgB	Lightowlers et al. [35]

*Echinococcus granulosus* 29 kDa	P-29	Gonzáles et al. [42]

*Echinococcus granulosus* paramyosin	EG36	Mühlschlegel et al. [68]

rEgG5	rEgG5	Lightowlers et al. [26]; Li et al. [69]

Thioredoxin peroxidase	TPx	Salinas et al. [70]; Margutti et al. [67]

EgA31	EgA31	Fu et al. [71]

Elongation factor 1*β*/*δ*	EgEF-1 *β*/*δ*	Margutti et al. [61]

Cyclophilin	EA21	Ortona et al. [63]

EpC1	EpC1	Li et al. [72]

Tropomyosin	Trp	Esteves et al. [73]

Heat shock protein 70	HSP70	Ortona et al. [64]

*Echinococcus granulosus* Tegumental antigen	EgTeg	Ortona et al. [65]

Eg19	Eg19	Delunardo et al. [66]

Heat shock protein 20	HSP20	Vacirca et al. [74]
